# Dietary genistein supplementation for breeders and their offspring improves the growth performance and immune function of broilers

**DOI:** 10.1038/s41598-018-23530-z

**Published:** 2018-03-26

**Authors:** Zengpeng Lv, Hao Fan, Beibei Zhang, Kun Xing, Yuming Guo

**Affiliations:** State key Laboratory of Animal Nutrition, College of Animal Science and Technology, China Agricultural University, 2 Yuanmingyuan West Road, Beijing, 100193 P. R. China

## Abstract

Genistein (GEN) is mainly extracted from soy plants and has potential functions as an antioxidant and in promoting immune function and growth. This study evaluated the effects of feeding breeders and their offspring dietary GEN on the immune function and growth performance of broiler chicks. Breeders were assigned to a control diet or GEN diet (control diet +400 mg/kg GEN), and their offspring were fed a control diet or GEN diet (control diet +40 mg/kg GEN). GEN treatment increased the body weight gain, tibial length, tibial width and slaughter performance of broilers and decreased the feed conversion ratio. The treatment also affected skeletal muscle myosin assembly and growth and increased growth hormone levels and IGF-I and IGFBP1 expression. Following GEN treatment, antigen processing and presentation, macrophage activation, B lymphocyte, NK cell and helper T cell proliferation, and CD4+ T lymphocyte differentiation all increased significantly. Increases were also observed in IgM and IgG concentrations, antibody titers, and antioxidant capacity. In addition, GEN treatment activated the Toll-like receptor signaling pathway and MAPK cascade signaling pathway. In summary, dietary GEN supplementation for breeders and their offspring can improve the growth performance and immune function of broiler chicks.

## Introduction

Isoflavones (ISFs), including genistein (GEN), daidzein and glycitein, are widely found in soy plants and have potential functions as antioxidants and in immune function and detoxification^1^. GEN has shown positive effects on breast cancer, prostate cancer, postmenopausal syndrome, osteoporosis, and cardiovascular disease^2^.

In recent years, ISFs, especially GEN, have been widely used in livestock and poultry production. Studies show that ISFs improve animal growth and reproductive performance and the quality of animal products^3–6^. For example, studies have shown that GEN could increase the feed intake and weight gain of pigs^7^, as well as increase the slaughter performance, carcass length, hind leg mass and carcass lean percentage of piglets^8^. It has also been demonstrated that feeding broilers ISFs increases their weight gain, feed intake and meat quality^9^, as well as the feed conversion and breast muscle rate^10^.

GEN is an important member of the multifarious group of phytoestrogens, which have been indicated to precisely orchestrate processes related to immune function^2^, increase the number of B cells in peripheral blood, and reduce oxidative DNA damage^11^. Furthermore, GEN supplementation has been shown to promote thymus development in adult mice^12^. Similar results were also found in *in vitro* studies in which GEN treatment had a protective effect on DNA and inhibited oxidative damage in lymphocytes^13^. In contrast, high doses of GEN have been shown to inhibit the immune response^14^.

Maternal nutritional status can affect offspring weight through effects on metabolism. In addition, early development plays an important role in the growth of chicks. For poultry, maternal effects are direct (resource supply) or indirect (genetic inheritance). Interestingly, ISFs have been reported to be deposited in eggs^15^. Moreover, studies have revealed that feeding adult mice GEN could induce genetic modification of DNA and affect the metabolic status of their offspring^16^. GEN treatment can increase natural killer (NK) cell activity and antibody levels in adult mice and their offspring^17^. Poultry development is unique. After fertilization, broilers are developed in eggs from 1 to 21 days of embryonic age, during which the main source of nutrition is the nutrients in eggs. After hatching, to 42 days, broilers get their nourishment mainly through feeding. Studies about GEN for broilers have mostly considered the period from 1 to 42 days of age, ignoring embryonic effects of GEN. In addition, an understanding of the effects of how GEN regulates broiler development from fertilization to slaughter is lacking. Therefore, we fed both breeder hens and broilers GEN, so broilers were regulated by GEN throughout development. The present study was conducted to determine whether dietary GEN supplementation for breeders and their offspring can affect embryonic development and improve growth performance and immune function in broiler chicks.

## Results

### Effects of GEN treatment on growth performance

The effects of GEN treatment on the growth performance of broilers are shown in Table 1. The results show that GEN treatment increased (P < 0.05) the body weight gain and induced a tendency for increased tibial width (P = 0.051) of broilers by day 21 but reduced the feed conversion ratio (P < 0.05) and had no significant effect on feed intake. In addition, there was an increase (P < 0.05) in liver, heart and thymus index values during the early growth of GEN-treated broilers compared with values for the control (CON) group, but there was no significant effect on other organ indices. In addition, for broilers at 42 days of age, GEN treatment tended to increase (P = 0.088) body weight gain and reduce the feed conversion ratio (P < 0.05). In addition, the breast muscle rate and leg muscle rate of the GEN group were significantly higher than those of the CON group (P < 0.05), and there was no significant effect on the dressing percentage (P = 0.161). However, the abdominal fat percentage decreased significantly (P < 0.05) after GEN treatment.

**Table 1:** Effects of dietary supplementation of broiler breeder hens and their offspring with genistein on broilers’ growth performance at 21 and 42 days of age.

	CON	GEN	P-value
Day 21			
BWG	0.791 ± 0.020	0.815 ± 0.016	0.019
FI	1.077 ± 0.046	1.047 ± 0.064	0.302
FCR	1.37 ± 0.04	1.31 ± 0.06	0.048
TL	75.26 ± 1.20	76.76 ± 1.67	0.102
TW	13.08 ± 0.61	13.78 ± 0.48	0.051
TS	267 ± 31	291 ± 31	0.292
HI	0.47 ± 0.03	0.55 ± 0.05	0.017
LI	2.07 ± 0.24	2.45 ± 0.25	0.024
SI	0.10 ± 0.05	0.08 ± 0.02	0.241
BI	0.16 ± 0.03	0.15 ± 0.03	0.811
TI	0.40 ± 0.05	0.53 ± 0.08	0.006
Day 42			
BWG	2.80 ± 0.06	2.87 ± 0.07	0.088
FI	4.74 ± 0.14	4.68 ± 0.15	0.446
FCR	1.68 ± 0.03	1.63 ± 0.04	0.011
DP	74.05 ± 1.46	74.92 ± 1.17	0.161
AFP	1.69 ± 0.14	1.45 ± 0.18	0.008
BMR	15.25 ± 0.59	16.33 ± 1.13	0.031
LMR	21.30 ± 0.87	22.22 ± 0.69	0.034

BWG, body weight gain (kg); FI, feed intake (kg); FCR, feed conversion ratio; TL, tibia length(mm); TW, tibia width (mm); TS, tibia strength (kg/cm2); DP, dressing percentage (%); AFP, abdominal fat percentage (%); BMR, breast muscle rate (%); LMR, leg muscle rate (%); HI, heart index (%); LI, liver index (%); SI, spleen index (%); BI, bursa index (%); TI, thymus index (%); CON, control group; GEN, genistein group. The data were expressed as the mean ± SD (n = 8 broiler chickens except from BW, FI and FCR for which n = 8 replicate cages), with a P-value.

### Effects of GEN treatment on serum hormone levels

The effects of GEN treatment on serum hormone levels are listed in Table 2. The data show that GEN-treated broilers had higher (P < 0.05) serum growth hormone (GH) levels than those in the CON group at 21 days of age. In addition, a reduction (P < 0.05) in serum levels of estradiol (E2) was observed in the GEN-treated group, but GEN treatment had no significant effects on triiodothyronine (T3) or tetraiodothyronine (T4) levels.

**Table 2:** Effects of dietary supplementation of broiler breeder hens and their offspring with genistein on broilers’ hormone levels in the serum at 21 days of age.

	T3	T4	GH	E2
Diet				
CON	1.04 ± 0.36	55.8 ± 5.93	1.13 ± 0.12	32.6 ± 8.21
GEN	1.07 ± 0.21	55.0 ± 8.05	1.39 ± 0.06	25.0 ± 4.72
*P*-value	0.873	0.82	<0.001	0.038

T3, triiodothyronine (ng/mL); T4, thyroxine (ng/mL); GH, growth hormone (ng/mL); E2, estradiol (pg/mL); CON, control group; GEN, genistein group. The data are expressed as the mean ± SD (n = 8 broiler chickens), with a P-value.

### Effects of GEN treatment on immune system parameters

**Figure 1:**
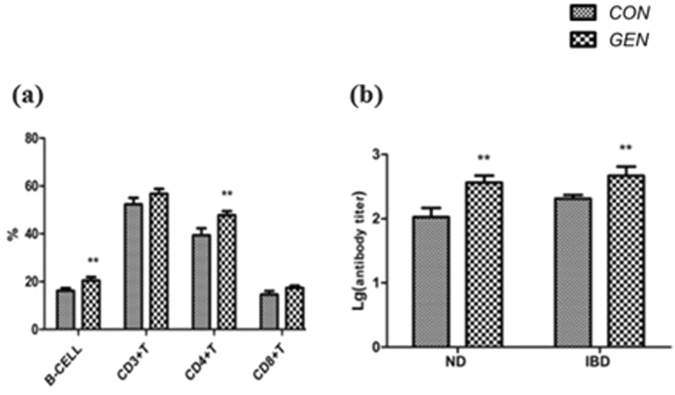
Effects of dietary supplementation of broiler breeder hens and their offspring with genistein on broilers’ peripheral blood lymphocytes and antibody titers. (**a**) Effects of GEN treatment on peripheral blood lymphocyte percentages of broilers at 21 days of age. (**b**) Effects of GEN treatment on the antibody titers for Newcastle disease (ND) and infectious bursal disease (IBD) viruses in the serum of broilers at 21 days of age. CON, control group; GEN, genistein group. Data are expressed as the mean ± SD (n = 8 replicates). “**”represents significant differences (P < 0.05).

The effects of GEN treatment on immune system parameters, including peripheral blood lymphocyte classification, lymphocyte proliferation, serum immunoglobulin (Ig) levels, and serum antibody levels, are listed in Fig. 1 and Table 3. As shown in Fig. 1a, GEN treatment significantly increased the ratio of B cells (P < 0.05) and CD4+ T cells (P < 0.05) in the peripheral blood of broilers at 21 days of age but had no significant effects on CD3+ T or CD8+ T cells. In addition, GEN treatment resulted in an increase in the proliferative responses of T cells (P = 0.070) and B cells (P = 0.102), as evidenced by the lack of changes in concanavalin A (ConA) and lipopolysaccharide (LPS) stimulation index (SI) values *in vitro* using the peripheral blood from broilers at 21 days of age (Table 3). Furthermore, GEN treatment increased the serum levels of IgM (P = 0.015) and IgG (P = 0.078) but had no significant effect on IgA levels (Table 3). In addition, as shown in Fig. 1b, GEN treatment significantly increased the serum antibody titers for Newcastle disease (ND) and infectious bursal disease (IBD) (P < 0.05) compared with titers in the CON group in broilers at 21 days of age.

**Table 3:** Effects of dietary supplementation of broiler breeder hens and their offspring with genistein on broilers’ lymphocyte proliferation *in vitro* and immunoglobulin levels in the serum at 21 days of age.

	LPS SI^a^	CONA SI^b^	IgM (ug/mL)	IgG (ng/mL)	IgA (ug/mL)
Diet					
CON	1.37 ± 0.12	1.23 ± 0.13	188 ± 51	41.73 ± 13.23b	435 ± 69
GEN	1.52 ± 0.21	1.38 ± 0.12	256 ± 41	56.84 ± 10.71a	457 ± 67
*P*-value	0.102	0.070	0.015	0.078	0.360

We induced T and B lymphocyte cell multiplication in peripheral blood lymphocytes through concanavalin A and lipopolysaccharide stimulation, respectively. ^a^Lipopolysaccharide stimulus index; ^b^concanavalin A stimulus index. CON, control group; GEN, genistein group. Data are expressed as the mean ± SD (n = 8 broiler chickens), with a P-value.

### Effects of GEN treatment on antioxidant indices

The effects of GEN treatment on antioxidant indices are listed in Table 4. Compared with levels in the CON group, GEN treatment increased (P < 0.05) the levels of alkaline phosphatase and metallothionein (MT) in the serum of 21-day-old broilers, and it increased the activity of total superoxide dismutase (T-SOD) and glutathione peroxidase (GSH-Px) and the total antioxidant capacity (T-AOC) of the liver. Additionally, GEN treatment reduced the content of malondialdehyde (MDA) in the liver. However, no effect on catalase (CAT) activity in the liver was detected. Therefore, dietary GEN supplementation for breeders and their offspring can improve antioxidant capacity in broiler chicks.

**Table 4:** Effects of dietary supplementation of broiler breeder hens and their offspring with genistein on broilers’ antioxidant enzyme activities at 21 days of age.

	AKP	T-SOD	GSH-Px	CAT	T-AOC	MDA	MT
Diet							
CON	1657 ± 618	295 ± 48	23.05 ± 2.73	14.77 ± 3.05	0.60 ± 0.20	0.94 ± 0.17	7.99 ± 0.39
GEN	2631 ± 592	387 ± 63	31.43 ± 6.14	16.05 ± 4.43	0.98 ± 0.25	0.76 ± 0.19	8.53 ± 0.21
P-value	0.006	0.008	0.003	0.479	0.007	0.095	0.015

AKP, alkaline phosphatase in serum (U/L); T-AOC, total antioxidant capacity of the liver (U/mg prot); MDA, malondialdehyde in the liver (mmol/mg prot); CAT, catalase in the liver (U/mg prot); T-SOD, total superoxide dismutase in the liver (U/mg prot); GSH-Px, glutathione peroxidase in the liver (U/mg prot). MT, metallothionein (ng/mL); CON, control group; GEN, genistein group. Data are expressed as the mean ± SD (n = 8 broiler chickens), with a P-value.

### Effects of GEN treatment on RNA-seq statistics

**Figure 2:**
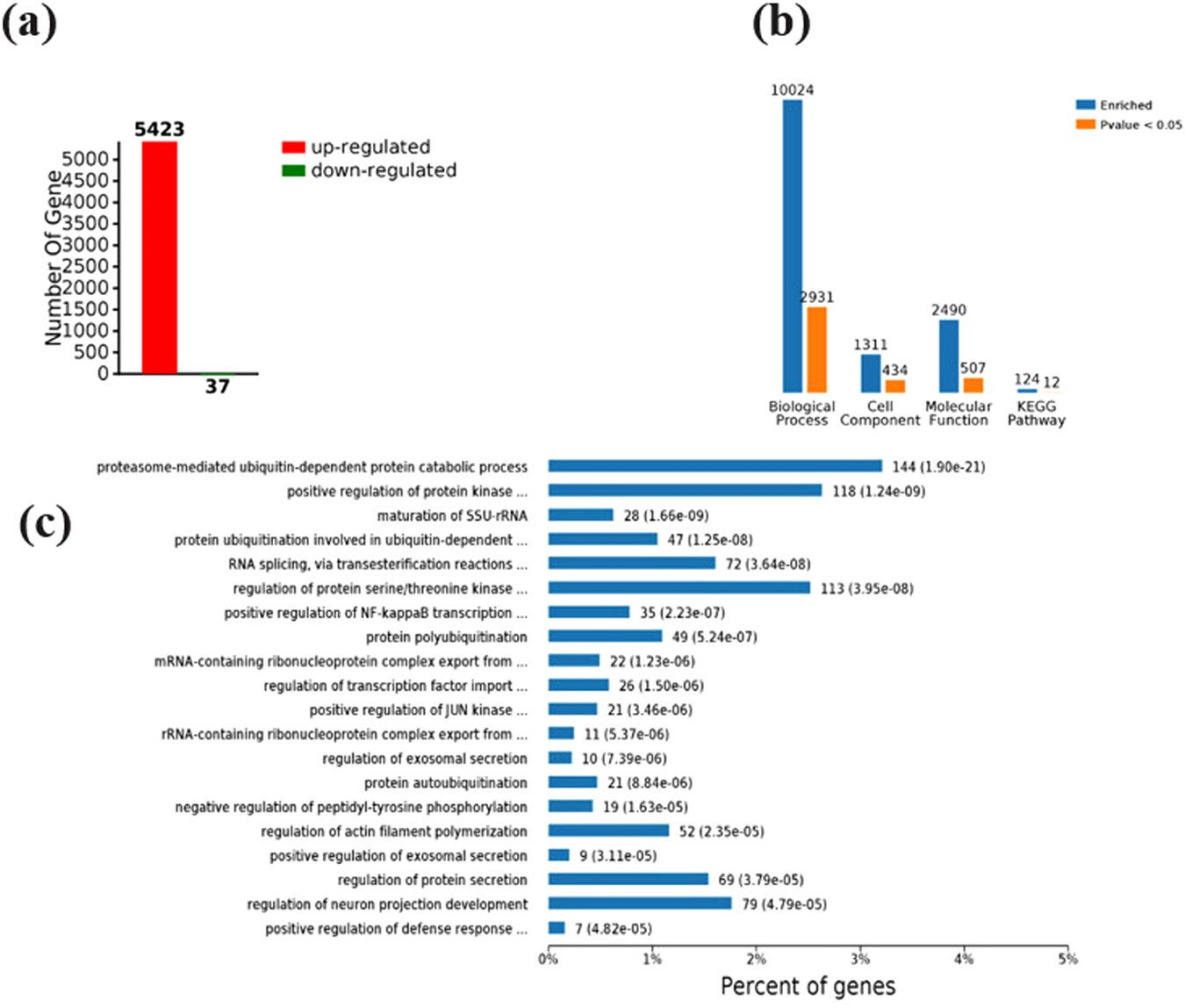
Effects of dietary supplementation of broiler breeder hens and their offspring with genistein on broilers’ differential gene expression at 21 days of age. (**a**) The number of differentially expressed genes (DEGs) (up/down-regulated) after GEN treatment. (**b**) The number of DEGs enriched in GO (Biological Process, Cell Component, Molecular Function) and KEGG Pathway terms. (**c**) The top twenty significantly enriched biological processes.

The effects of GEN treatment on RNA-Seq results are shown in Table 5 and Fig. 2. In this study, we established eight cDNA libraries from the livers of the broilers in the CON and GEN groups, with four replicates for each group. The RNA-Seq analysis was generated from 77,145,380 to 88,858,140 raw reads for each library, with an average of 83,529,344 and 82,280,519 paired-end reads for the CON and GEN groups, respectively. After filtering out low-quality reads, the average numbers of clean reads were 79,268,807 (94.89%) and 77,883,472 (94.65%) for the CON and GEN groups, respectively. The clean reads were used for all further analyses. After assembly, 13,888 mRNA sequences were obtained from the two groups. Approximately 83.0% of the reads in each library were uniquely mapped to the galGal4 assembly of the chicken genome, and the average mapping rates were 83.15% and 82.75% for the CON and GEN groups, respectively (Table 5). As Fig. 2a shows, 5450 differentially expressed genes (DEGs, 5423 up-regulated and 37 down-regulated) were identified between the CON group and GEN group (false discovery rate (FDR) ≤ 0.05, fold-change ≥1.3 or ≤0.5) using Cuffdiff software. The numbers of significantly enriched terms in biological process, cell component, molecular function and KEGG pathway were 2931, 434, 507 and 12, respectively (Fig. 2b). The top twenty significantly enriched terms are shown in Fig. 2c. The results indicate that GEN treatment enhanced protein ubiquitination, positively regulated NF-kappa B transcription factor activity, and stimulated the defensive response to external stimuli.

**Table 5:** RNA-seq results for samples used in the experiment.

Sample	Original reads numbers	Q30%	Mean Quality Score (PF)	Clean read numbers	Reads1 numbers	Reads2 numbers	Overall match rate (%)
CON1	77183076	91.40	35.85	73484896	31253727	31216284	85.0
CON2	82345376	91.38	35.84	78284742	30945198	30945198	79.1
CON3	87264386	91.01	35.75	82670180	33934514	33934514	85.8
CON4	87324538	91.47	35.86	82635412	34183206	34183206	82.7
GEN1	88858140	91.33	35.88	84048930	34099771	34135451	81.1
GEN2	77145380	90.89	35.61	72936664	28884332	28884332	79.2
GEN3	84951950	91.65	35.49	80384550	33876783	33876783	84.3
GEN4	78166606	91.77	35.86	74163744	32078315	32042937	86.4

CON, control group; GEN, genistein group. Control group samples: CON1-4; GEN group samples: GEN1-4.

### Effects of GEN treatment on the functional categorization of DEGs and pathway analysis in relation to growth and development

**Figure 3:**
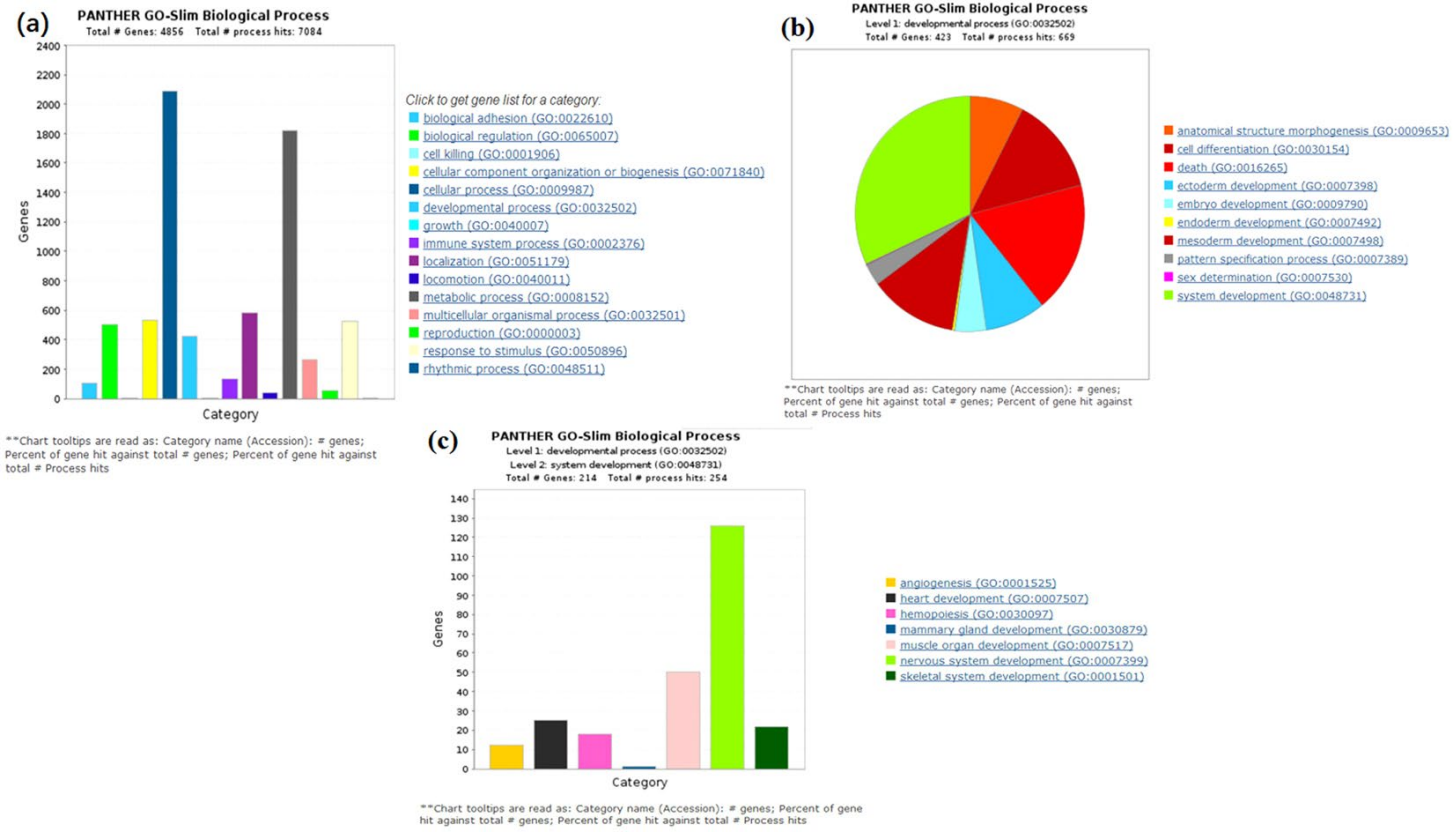
Effects of dietary supplementation of broiler breeder hens and their offspring with genistein on broilers’ differentially expressed genes (DEGs) for functional categorization and pathway analysis related to growth and development. (**a**) Panther Go-Slim Biological Processes based on 4856 DEGs. (**b**) Panther Go-Slim Biological Processes based on 423 DEGs clustered with the term developmental process (GO:0032502). (**c**) Panther Go-Slim Biological Processes based on 214 DEGs clustered with the term system development process (GO:0048731).

The effects of GEN treatment on the functional categorization of DEGs and pathway analysis in relation to growth and development are listed in Fig. 3. We used the Panther Classification System (http://www.pantherdb.org/) to perform a biological process analysis of the DEGs. As shown in Fig. 3a, the major DEGs were mainly enriched in the terms cellular process, metabolic process, localization, reproduction and response to stimulus, with 423 DEGs enriched in the terms developmental process and growth, which indicates that the GEN treatment affected the growth and development of broilers. In addition, the fact that some DEGs were related to cell differentiation, ectoderm development, embryo development, endoderm development, mesoderm development, and system development further supports the idea that GEN treatment affected the growth and development of broilers (Fig. 3b). Furthermore, as shown in Fig. 3c, 50 DEGs were enriched in muscle development, and the mRNA levels of 12 myosin-associated genes (MYO1E, MYO1F, MYO5A, MYO18A, MYH11, MYH9, MYO9B, MYO10, MYO9A, MYO1D, MYH10, MYO1A) were up-regulated in the GEN group, as were those of 8 cadherin-related genes (CDH3, PCDH1, DCHS1, CDH2, CDH17, CDH1, CDH4, CDH13), 2 muscleblind-like (MBNL) genes (MBNL1, MBNL3), and 4 PDZ and LIM domain-related (PDLIM) genes (PDLIM4, PDLIM5, PDLIM7, PDLIM1). In addition, DLL1|1.37, which promotes bone growth; MYH11|1.50, which participates in myosin thick filament assembly; TGFBR2|2.03 and THBS1|1.60, which regulate the development of growth-plate cartilage; and CHD7|1.67 and RAB1A|1.42, which promote GH secretion, were all up-regulated.

### Effects of GEN treatment on the functional categorization of DEGs and pathway analysis in relation to immune function

**Figure 4:**
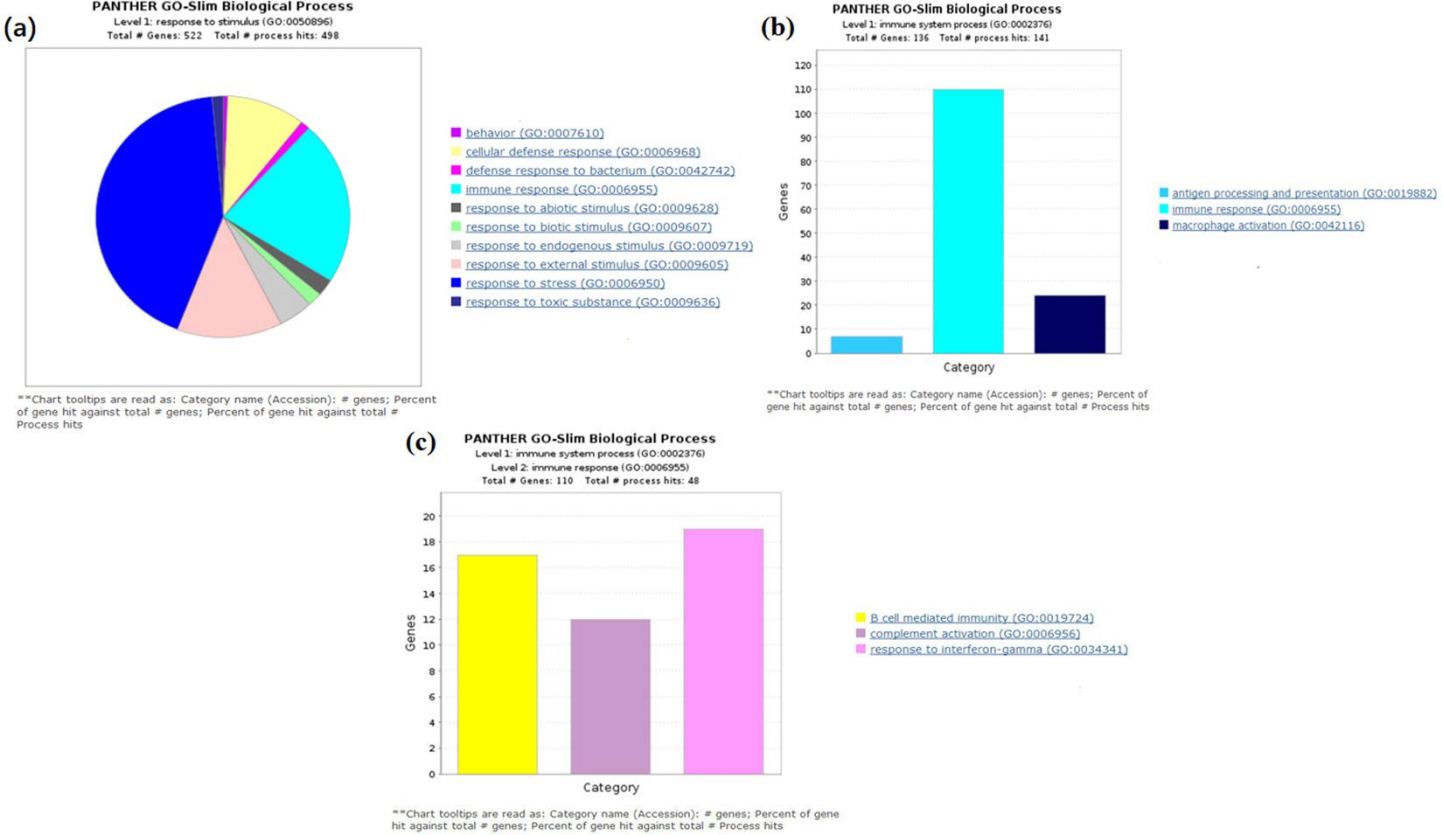
Effects of dietary supplementation of broiler breeder hens and their offspring with genistein on broilers’ DEGs for functional categorization and pathway analysis related to immune function. (**a**) Panther Go-Slim Biological Processes based on 522 DEGs clustered with the term response to stimulus (GO:0050896). (**b**) Panther Go-Slim Biological Processes based on 136 DEGs clustered with the term immune system process (GO:0002376). (**c**) Panther Go-Slim Biological Processes based on 110 DEGs clustered with the term immune response (GO:00069555).

The effects of GEN treatment on the functional categorization of DEGs and pathway analysis in relation to immune function are shown in Fig. 4 and Table 6. The results show that 522 and 136 DEGs were enriched in the terms response to stimulus (GO:0050896) and immune system process (GO:0002376), respectively, with the majority of genes enriched in the following terms: cellular defense response, defense response to bacterium, immune response, response to endogenous stimulus, response to external stimulus and response to stress (Fig. 4a). Furthermore, the DEGs enriched in the immune system process were related to antigen processing and presentation (GO:0019882), MHC class II (GO:0002504), and macrophage activation (GO:0042116) (Fig. 4b). In addition, DEGs enriched in the immune response included those related to B cell-mediated immunity (GO:0019724), complement activation (GO:0006956) and response to interferon-gamma (GO:0034341) (Fig. 4c). We selected the immune-related DEGs using OmicsBean software for deep-level GO clustering (10 levels) analysis (Table 6). The immune-related DEGs were mainly enriched in the following terms: activating the Toll-like receptor signaling pathway, NF-kappa B signaling activity, promoting NK cell and helper T cell differentiation, inhibiting T cell apoptosis, activating B cell receptor signaling, and promoting B cell proliferation and secretion of Ig. Thus, GEN treatment activated type I interferon signaling and improved the anti-viral capacity of broilers. In summary, these results show that dietary GEN supplementation for breeders and their offspring up-regulates the expression of immune-related genes in broilers at 21 days of age.

**Table 6:** Effects of dietary supplementation of broiler breeder hens and their offspring with genistein on broilers’ DEGs for GO clustering analysis related to the immune system.

GO Name	*P*-value	Genes|Fold change	Count	Pop Hit
positive regulation of NF-kappa B transcription factor activity	<0.0001	CIB1|1.75;PRKCB|1.75;CARD11|1.86;TRAF2|1.87;SPHK1|1.95;CD40|2.54;IKBKB|1.85;CHUK|1.75;RNF25|1.83;CTH|1.8914;DDRGK1|1.67;TLR4|1.81;MYD88|1.56;EDA|1.62;TIRAP|1.86;TRIM14|1.98;IRAK2|1.56;EDA2R|1.93;TICAM1|1.56;PSMA6|1.49;TRIM37|1.53;TRIM25|1.52;RPS6KA5|1.50;RIPK4|1.48;NOD1|1.53;WNT5A|2.33;MAP3K13|1.39;MTPN|1.38;FER|1.41;IL1RAP|1.39;TNFRSF11A|1.38;TRAF6|1.40;RIPK1|1.38;UBE2N|1.35;CD40LG|1.90	35	81
positive regulation of defense response to virus by host	<0.0001	SIN3A|1.75;SELK|1.77;CREB3|2.32;MAVS|1.751;TRAF3IP2|1.46;SMURF1|1.48;MB21D1|1.77	7	8
MyD88-dependent toll-like receptor signaling pathway	0.0002	CHUK|1.75;NFKBIA|1.65;NFKB1|1.64;TLR4|1.80;MYD88|1.56;TIRAP|1.86;HSPD1|1.70;IRAK2|1.55;IRF1|1.52;ENSGALG00000009237|1.51;JUN|1.53;MAP2K2|1.488;TRAF6|1.40;TLR7|1.60;IRF-3|1.41;TLR1B|1.45	16	35
toll-like receptor 4 signaling pathway	0.0006	CHUK|1.75;PELI1|1.61;NFKBIA|1.66;NFKB1|1.65;TLR4|1.81;MYD88|1.56;TIRAP|1.86;LYN|1.58;JUN|1.53;HMGB1|1.48;MAP2K2|1.50;DAB2IP|1.40;TRAF6|1.40;PIK3AP1|1.38	14	31
positive regulation of type I interferon-mediated signaling pathway	0.0008	FADD|1.83;MAVS|1.75;NLRC5|1.60;LSM14A|1.41;WNT5A|2.33;IRF-3|1.41	6	8
toll-like receptor signaling pathway	0.0008	TNFAIP3|1.93;CHUK|1.75;PELI1|1.61;CACTIN|1.68;NFKBIA|1.65;NFKB1|1.64;TNIP2|1.89;TLR4|1.80;RPS6KA3|1.55;MYD88|1.56;TIRAP|1.86;HSPD1|1.70;IRAK2|1.55;LYN|1.58;IRF1|1.52;TICAM1|1.56;MAPKAPK2|1.48;ENSGALG00000009237|1.51;JUN|1.53;HMGB1|1.48;MAP2K2|1.48;MAPKAPK3|1.402;DAB2IP|1.40;TRAF6|1.40;PIK3AP1|1.37;TLR7|1.60;IRF-3|1.41;TLR1B|1.45	28	84
toll-like receptor 5 signaling pathway	0.0008	TNFAIP3|1.93;CHUK|1.75;NFKBIA|1.65;NFKB1|1.64;MYD88|1.56;TIRAP|1.86;JUN|1.53;MAP2K2|1.48;TRAF6|1.40	9	16
toll-like receptor 2 signaling pathway	0.0010	CHUK|1.75;NFKBIA|1.65;NFKB1|1.64;TNIP2|1.89;MYD88|1.56;TIRAP|1.86;LYN|1.58;ENSGALG00000009237|1.51;JUN|1.53;HMGB1|1.48;MAP2K2|1.488;TRAF6|1.40;PIK3AP1|1.37	13	29
toll-like receptor 21 signaling pathway	0.0024	CHUK|1.75;NFKBIA|1.65;NFKB1|1.64;MYD88|1.56;JUN|1.53;MAP2K2|1.4881;TRAF6|1.4034	7	12
toll-like receptor 15 signaling pathway	0.0025	CHUK|1.75;NFKBIA|1.65;NFKB1|1.648;MYD88|1.56;TIRAP|1.86;JUN|1.53;MAP2K2|1.48;TRAF6|1.40	8	15
positive regulation of B cell proliferation	0.0033	CARD11|1.86;CD40|2.53;SASH3|2.01;TFRC|1.91;NCKAP1L|1.97;PELI1|1.61;TLR4|1.80;TICAM1|1.56;MSH6|1.48;CDKN1A|1.66;ATAD5|1.49;PMS2|1.48	14	36
regulation of T cell apoptotic process	0.0033	TSC22D3|1.88;FADD|1.83;GPAM|2.180;SIGLEC1|2.10;DOCK8|1.57;VHL|1.54;PDCD1|2.67;CASP8|1.56;WNT5A|2.37;SLC46A2|1.99	10	22
negative regulation of lymphocyte apoptotic process	0.0037	TSC22D3|1.88;FADD|1.84;GPAM|2.18;NOC2L|1.63;DOCK8|1.57;VHL|1.55;IRS2|0.60;SLC46A2|2.00;MIF|1.38	9	19
toll-like receptor 7 signaling pathway	0.0037	CHUK|1.75;NFKBIA|1.65;NFKB1|1.64;MYD88|1.56;JUN|1.53;MAP2K2|1.481;TRAF6|1.40;PIK3AP1|1.37;TLR7|1.60	9	19
T-helper cell differentiation	0.0058	STAT3|2.08;ANXA1|2.93;RC3H2|1.54;ATP7A|1.53;RC3H1|1.52;IL18R1|1.85;STAT6|1.51;HMGB1|1.48;SOCS5|1.47;BATF|2.1;PRKCZ|1.37	11	27
negative regulation of activation-induced cell death of T cells	0.0063	TSC22D3|1.88;FADD|1.84;GPAM|2.180	3	3
immunoglobulin secretion	0.0072	TRAF2|1.8;CD40|2.53;TRAF3IP2|1.46;TRAF6|1.40;CD40LG|1.90	5	8
activation of NF-kappaB-inducing kinase activity	0.0084	CARD11|1.86;TRAF2|1.8;CHUK|1.75;NFKBIA|1.65;NFKB1|1.64;TIRAP|1.86;COPS8|1.46;TRAF6|1.40;MAP3K7|1.36	9	21
negative regulation of T cell apoptotic process	0.0130	TSC22D3|1.88;FADD|1.83;GPAM|2.18;DOCK8|1.57;VHL|1.54;SLC46A2|1.98	6	12
regulation of toll-like receptor 4 signaling pathway	0.0138	PELI1|1.61;TIRAP|1.86;LYN|1.58;HMGB1|1.48;DAB2IP|1.40	5	9
secretion of lysosomal enzymes	0.0216	LYST|1.58;LXR|1.42;GNPTAB|1.34	3	4
regulation of activation-induced cell death of T cells	0.0216	TSC22D3|1.88;FADD|1.83;GPAM|2.18	3	4
T-helper 1 cell differentiation	0.0235	ANXA1|2.93;IL18R1|1.85;STAT6|1.51;HMGB1|1.48;SOCS5|1.47	5	10
positive regulation of CD4-positive, alpha-beta T cell differentiation	0.0303	CD83|1.81;SASH3|2.01;NCKAP1L|1.97;ANXA1|2.93;SOCS5|1.47;PRKCZ|1.37	6	14
regulation of MyD88-dependent toll-like receptor signaling pathway	0.0339	IRF1|1.52;IRF-3|1.41	2	2
regulation of B cell receptor signaling pathway	0.0366	PRKCB|1.74;PTPN6|1.57;PTPRC|1.56;LYN|1.58;PLCL2|1.50	5	11
regulation of toll-like receptor signaling pathway	0.0462	TNFAIP3|1.93;PELI1|1.61;CACTIN|1.68;TIRAP|1.86;LYN|1.58;IRF1|1.52;HMGB1|1.48;DAB2IP|1.40;PIK3AP1|1.37;IRF-3|1.41	10	31
positive regulation of NK T cell differentiation	0.0465	TGFBR2|2.027;AP3D1|1.68;AP3B1|1.38	3	5
T-helper cell lineage commitment	0.0465	STAT3|2.08;STAT6|1.51;BATF|2.11	3	5
regulation of toll-like receptor 2 signaling pathway	0.0465	TIRAP|1.86;LYN|1.58;HMGB1|1.48	3	5

### Effects of GEN treatment on pathway analysis

**Figure 5:**
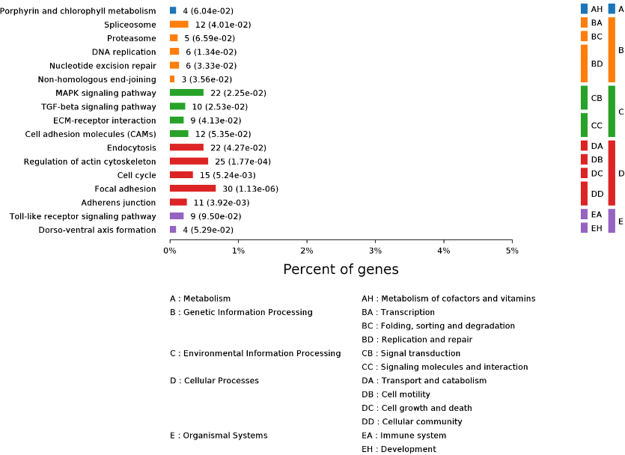
Effects of dietary supplementation of broiler breeder hens and their offspring with genistein on broilers’ DEGs for pathway analysis.

The results of GEN treatment on pathway analysis are shown in Fig. 5. Analyses of the functional and signaling pathway enrichment of DEGs were conducted using the KEGG PATHWAY website. The DEGs were mainly enriched in the following terms: porphyrin and chlorophyll metabolism, DNA replication, nucleotide excision repair, and cell cycle signaling pathway, as well as MAPK signaling pathway, TGF-beta signaling pathway, ECM-receptor interaction, adherens junction, and Toll-like receptor signaling pathway, which are important for regulating signal transmission and immune function.

## Discussion

GEN has beneficial effects related to immunomodulation^18,19^ and anti-oxidation^20,21^, as well as growth-promoting effects in mammals^22,23^. However, the effects of supplying dietary GEN to breeder hens and their offspring on the growth performance and immune function of broilers have not been reported. Here, we demonstrate that dietary GEN supplementation for breeders and their offspring has effects on embryonic development, growth performance and immune function in broiler chicks.

As reported previously, the dietary addition of ISFs can affect average daily gain and average daily feed intake and may affect carcass traits in some broilers^10^. In this study, we confirmed the positive effects of GEN supplementation on the growth performance of broilers, including improved body weight gain and reduced feed conversion ratio at 21 and 42 days of age. Over the past few years, benefits for growth performance in chickens have been reported with the addition of 10 to 20 mg/kg GEN, which has been shown to increase the body weight gain of broilers and reduce the feed conversion ratio^9^. GEN treatment improved the carcass traits of broilers at 42 days of age, such as breast muscle rate and leg muscle rate, and reduced the abdominal fat percentage. Our results are consistent with those of studies showing that dietary GEN can increase breast muscle weight in poultry^24^.

Embryo development, muscle development and bone growth are extremely important for the growth performance of broilers^25–27^. Myosin, cadherin, MBNL proteins, and PDLIM proteins are all key factors in muscle development that can increase growth performance when their expression is increased^28,29^. In the present study, we observed that GEN treatment induced the differential expression of 423 genes enriched in terms of developmental process and growth compared with expression in the CON group. These genes mainly affect cell differentiation, ectoderm development, embryo development, endoderm development, mesoderm development and system development^30^. In addition, genes for myosin-associated proteins (MYO1E, MYO1F, MYO5A, MYO18A, MYH11, MYH9, MYO9B, MYO10, MYO9A, MYO1D, MYH10, MYO1A) were up-regulated in the GEN-treated group, as were 8 cadherin-related genes (CDH3, PCDH1, DCHS1, CDH2, CDH17, CDH1, CDH4, CDH13), 2 MBNL genes (MBNL1, MBNL3), and 4 PDLIM genes (PDLIM4, PDLIM5, PDLIM7, PDLIM1). In addition, GEN treatment increased the mRNA expression of delta-like canonical Notch ligand 1 (DLL1), transforming growth factor beta receptor (TGFBR) and thrombospondin 1 (THBS1), which regulate the development of growth plate cartilage; these findings are consistent with previous results^31^.

The GH axis plays a crucial role in the regulation of animal growth^32^. When pituitary-derived ghrelin binds to GH receptors in the liver, GH levels increase, as does the expression of GH receptors and insulin-like growth factor I (IGF-I)^33^. IGFs play an important role in receptor-mediated mitosis and the regulation of IGF binding proteins (IGFBPs)^34^, which promote DNA synthesis and cell proliferation through the inhibition of cell apoptosis. Thus, IGF activation can promote animal growth^35^. In our study, after GEN treatment, we found that serum GH levels in broilers at 21 days of age were significantly increased, as was the mRNA expression of CHD7 and RAB1A in the liver, which participate in the regulation of GH secretion. Additionally, dietary GEN up-regulated the mRNA expression of IGF-I and IGFBP1 in the livers of broilers. We also found that GEN treatment decreased serum E2 levels in broilers, which may be related to the role of GEN in feedback regulation of sex hormones, although the underlying mechanism remains to studied.

The thymus and bursa in poultry are central immune organs involved in lymphocyte differentiation and the immune response. Peripheral immune organs, which include lymph nodes and the spleen, play roles related to antigen immune responses. Studies have shown that adding GEN to feed can improve thymus function and the bursa index of poultry^36,37^; however, different findings have been described elsewhere^38^, and feeding perinatal cows different doses of GEN results in different effects on thymus weight in calves^39^. In this study, dietary GEN supplementation for breeders and their offspring had no significant effect on immune organ indices in broilers.

Poultry immune cells include lymphocytes, mononuclear phagocytes and antigen-presenting cells. The main role of lymphocytes, which include T lymphocytes and B lymphocytes, is to recognize antigens and activate the corresponding immune response^40^. Our data illustrated that dietary GEN supplementation for breeders and their offspring affected antigen processing and presentation, immune responses, and the activation of macrophages of broiler chicks at 21 days of age. In addition, OmicsBean analysis showed that GEN treatment activated the B cell receptor signaling pathway, inhibited T cell apoptosis, and promoted the proliferation of B cells and the differentiation of helper T cells, CD4+ T cells, and alpha-beta T cells of broilers. Accordingly, the proportions of B cells and CD4+ T cells in the peripheral blood of GEN-treated broilers were increased, as shown by flow cytometry analysis. These results are in agreement with the results of previous studies^12,41^. Furthermore, we used LPS and ConA to stimulate peripheral blood lymphocytes in broilers at 21 days of age, which showed that GEN treatment promoted the proliferation of peripheral blood lymphocytes. Estrogens have been shown to increase NK cell activity in mice^42^, and GEN treatment has been shown to increase the activity of NK cells both *in vivo* and *in vitro*^43,44^. Our results are consistent with the above findings as GEN treatment up-regulated the mRNA expression of TGFBR2, AP3D1, and AP3B1, which promote NK cell proliferation.

Studies have shown that GEN can enhance antioxidant, antimicrobial, anti-inflammatory and anti-cancer capacities in different species, leading to improvements to health and metabolic status^45,46^. Immunity-related molecules in cell membranes and blood may reflect regulatory immune function. IgG and IgM are important factors in the immune response and in preventing infection, and they can facilitate lysis and activate complements to promote phagocytosis^47^. Research shows that estrogen can increase IL-10 expression and the levels of IgM and IgG secreted by human mononuclear cells^48^. GEN also increases the Ig content of peripheral blood in a variety of animals^49,50^. We observed that GEN treatment for breeders and their offspring increased IgG and IgM levels in broilers at 21 days of age. The OmicsBean data also show that some immune-related DEGs were related to activating B cell receptor signaling, promoting B cell proliferation and Ig secretion. Furthermore, GEN treatment activated type I interferon signaling and improved the anti-viral capacity of broilers. We measured the antibody titers of broiler serum at 21 days of age, and the ND and IBD antibody titers in the GEN treatment group were significantly higher than those in the CON group. In addition, for broilers at 21 days of age, GEN treatment up-regulated the mRNA expression of LYST, LXR and GNPTAB, which are related to lysosomal secretion, and treatment significantly increased the serum levels of alkaline phosphatase.

The Toll-like receptor signaling pathway, NF-kappa B signaling pathway and MAPK signaling pathway are all critical for immunomodulation, which plays a role in activating immune cells and releasing immunity-related cytokines. These pathways can activate humoral immunity, cellular immunity and innate immune responses, leading to enhanced anti-inflammatory and anti-bacterial effects^51,52^. In our study, GO cluster analysis using DEGs between the GEN and CON groups showed that GEN treatment activated Toll-like receptor signaling, NF-kappa B signaling and MAPK signaling, with the up-regulation of MyD88 and TOLLIP mRNA expression, as well as of their downstream signaling factors. Our results agree with the results showing that GEN can increase the protein level of TLR2 in endometrial cells and enhance uterine immune function^53^. It has been reported that GEN can activate the ERK5 MAPK signaling pathway in breast cancer cells^54^ and significantly enhance LPS-stimulated MAPK signaling cascades in macrophages^55^. In the present experiment, dietary GEN supplementation for breeders and their offspring up-regulated the mRNA expression of c-FOS and c-JUN in the livers of chicks, which might affect cell proliferation and differentiation and apoptosis-mediated immune responses. These results suggest that dietary GEN supplementation for breeders and their offspring activates the Toll-like receptor signaling pathway, NF-kappa B signaling pathway and MAPK signaling pathway by up-regulating key genes related to immunomodulation and that dietary GEN treatment can improve immune function in chicks.

ROS production, which is increased by stress, can induce immunosuppression^56^. The phenolic hydroxyl groups of GEN can serve as hydrogen donors to combine with oxygen radicals. It has been reported that dietary GEN can also enhance the activity of antioxidant enzymes in the skin and intestines of mice^57^. In the current study, feeding laying broiler breeders and their offspring with GEN increased the activity of T-SOD, GSH-Px, and T-AOC in the livers of broilers, and it decreased the levels of MDA, which is a product of lipid peroxidation. In addition, transcriptome data showed that GEN treatment up-regulated the mRNA expression of SOD3 and MT4 in the liver. These findings are consistent with those of previous studies showing that GEN can improve T-SOD activity, GSH-Px activity and T-AOC capacity in rat livers, reducing lipid peroxidation^58^, and that GEN supplementation can up-regulate GSH-Px mRNA expression in cells^59^. Therefore, feeding breeders and offspring with GEN significantly increased the antioxidant capacity of broilers, which is favorable for improving the growth performance and immune function of broilers.

## Conclusion

We report, for the first time, that dietary GEN supplementation for breeders and their offspring improves the growth performance and immune function of broilers. The mechanisms of action underlying these effects are related to promoting muscle development and regulating GH secretion and to the antioxidant, anti-inflammatory and immunoenhancement effects of GEN. Dietary GEN supplementation for offspring activated Toll-like receptor signaling, NF-kappa B signaling and MAPK signaling in broilers; promoted the proliferation and differentiation of lymphocytes and NK cells; increased the levels of IgG and IgM and antibody titers; and improved the immune function of broilers. Moreover, GEN treatment up-regulated the levels of mRNA for myosin- and cadherin-related genes and increased GH levels. This all resulted in an increase in the growth performance of broilers. Therefore, dietary GEN supplementation for breeders and their offspring can improve the immune function and growth performance of broiler chicks. Treatment with GEN may provide an effective, safe and quick method for raising broilers and could reduce the use of the antibiotics, which have many potential adverse effects on broilers and on human health.

## Materials and Methods

### Ethics Statement

This study was carried out in strict accordance with the guidelines for the care and use of animals of China Agricultural University. All experimental procedures involving animals were approved by the Animal Care Commission of the College of Animal Science and Technology, China Agricultural University. Every effort was made to minimize animal pain, suffering, and distress and to reduce the number of animals used.

### Management of animals

The experiment was performed using laying broiler breeder hens (n = 8 replicate) and offspring broilers (n = 8 replicate) housed at a commercial farm (Zhuozhou, China) under standard conditions. After a 2-week acclimation period, a total of 480 57-week-old Ross 308 laying broiler breeder hens were allocated into two treatment groups (Ab and Bb); each treatment had 8 replicates, each replicate had 15 cages, and each cage had 2 laying broiler breeder hens. In addition, broiler breeder roosters were housed at a ratio of 30 hens to 1 male. Artificial insemination was performed at a ratio of 30 hens to 1 male, and we allowed 1 day of rest after 2 days of semen collection for males; artificial insemination was performed once every five days. The Ab and Bb groups were fed with a nutritionally balanced corn-miscellaneous meal (CSCM) diet with either no GEN or GEN added at 400 mg/kg for 8 weeks. CSCM diets were formulated to meet the nutrient requirements of laying broiler breeders according to the NRC guidelines (1994) (Table 7). Male breeders were caged and given a commercial diet. Hatching eggs (200 eggs per group) were collected in the final 3 days of the 8-week experimental period and then divided into two groups: A (CON) and B (GEN). Egg incubation was conducted under standard conditions with 70–80% humidity and at 37.8 °C. After hatching, chicks from each group were divided into 8 replicates, with 10 birds each. The chickens were housed in wire cages under a standard, gradually decreasing temperature regimen that ranged from 35 to 26 °C. The experiment lasted 6 weeks. Diets were provided ad libitum with constant access to water. Chickens were challenged with ND vaccine at day 7 and IBD vaccine at days 14 and 28. The birds from the CON group were fed the basal corn–soybean meal diet, which was formulated based on NRC guidelines (1994). The chicks from the GEN group were fed the same basal diet with the addition of 40 mg/kg GEN (Table 7).

**Table 7:** Composition and nutritional level of breeders and their offspring fed experimental diets (%).

Ingredient (%)	Breeders diets	Broilers diets (0–3 weeks)	Broilers diets (3–6 weeks)
Corn	68.99	53.28	57.59
Soybean meal	4	38.57	34.5
Corn protein	9.15	—	—
De-gossypol cottonseed protein	6	—	—
Limestone	7.76	1.05	1
Soybean oil	0.5	3.7	4
Dicalcium phosphate	2.09	1.98	1.67
NaCl	0.35	0.35	0.35
^a^Trace mineral premix	0.3	0.3	0.3
Choline chloride (50%)	0.12	0.3	0.25
Mycotoxin adsorbent	0.1	—	—
DL- Methionine	0.0515	0.22	0.14
^b^Vitamin premix	0.035	0.02	0.02
Santoquin	0.03	0.03	0.03
Phytase	0.016	—	—
4% Flavomycin	0.015	—	—
Lysine•HCl (8%)	0.373	0.12	0.05
Threonine	0.0664	—	—
Tryptophan	0.0481	—	—
Total	100	100	100
Avian metabolic energy MC/kg	2.83	2.9526	3.01
Crude protein (%)	16.1	21.6	20
Calcium (%)	3.48	1.051	0.96
Total phosphorus (%)	0.678	0.7	0.64
Available phosphorus (%)	0.47	0.45	0.4
Methionine (%)	0.34	0.5	0.4
Lysine (%)	0.805	1.15	1
Met + Cys (%)	0.626	0.86	0.74
Threonine (%)	0.6	0.8	0.74
Tryptophan (%)	0.18	—	—

^a^Broilers were supplied with the following nutrients (per kg complete diet): Cu, 8 mg; Zn, 75 mg; Fe, 80 mg; Mn, 100 mg; Se, 0.15 mg; I, 0.35 mg. ^b^Broilers were supplied with the following nutrients (per kg complete diet): vitamin A, 12500 IU; vitamin D3, 2500 IU; vitamin E, 30 IU; vitamin K3, 2.65 mg; thiamine, 2 mg; riboflavin, 6 mg; vitamin B12, 0.025 mg; biotin, 0.0325 mg; folic acid, 1.25 mg; pantothenic acid, 12 mg; niacin, 50 mg.

### Materials

The GEN used in this study was synthetically produced by the Kai Meng. Co (Xi An, China) Chemical Plant with a purity of 99.9%.

### Collection of samples and chemical analysis

At 21 days of age, one chick per replicate, with a body weight close to the average, was selected after 8 h of feed deprivation. One blood sample was collected from the wing vein of each chick in vacuum blood collection tubes, and the serum was centrifuged at 3000 × *g* for 15 min and stored at −20 °C until use for the detection of hormones, antibodies and Ig. Another blood sample was collected from the wing vein into vacuum blood collection tubes (with heparin sodium) to detect lymphocyte proliferation and percentages. Then, one chicken from each replicate was slaughtered. Liver samples for measurements of gene expression and antioxidative indices were immediately collected from 8 chickens in each group, and samples were frozen in liquid nitrogen and kept in a freezer (−80 °C).

### Carcass characteristics

One broiler from each replicate, with a live bodyweight close to the average of each replicate, was selected. Birds were weighed to the nearest gram, subjected to 24-h feed withdrawal with free access to water, reweighed and slaughtered by electrical stunning. The body after bleeding, without feathers, viscera, head and giblets was weighed, and the weight was expressed as a percentage of its live bodyweight and considered the dressing percentage. In addition, the weights of the breast, leg muscle, abdominal fat, liver (without gall bladder), heart, spleen, bursa, and thymus were recorded, and their relation to the live bodyweight of the birds, in percentages, was calculated.

### Radioimmunoassay for serum hormone concentrations

The serum levels of T3, T4, GH and E2 were measured using commercial double-antibody radioimmunoassay kits purchased from Shanghai Institute of Biological Products. The inter-assay coefficient of variation was 10%.

### Serum antibody and immunoglobulin levels

The serum antibody titers against ND and IBD viruses were determined using a commercial ELISA kit (IDEXX laboratories Inc., Westbrook, Maine, USA) according to the manufacture’s protocol, as were serum IgM, IgG and IgA levels.

### Lymphocyte proliferation

Peripheral blood mononuclear cells (PBMCs) were isolated using Ficoll density centrifugation^60^. Briefly, heparinized blood was diluted with Hank’s balanced salt solution at a ratio of 1:1 (no calcium, no magnesium, Life Technologies) and carefully layered on the top of Histopaque 1077 (Sigma-Aldrich Corporation) in a 10-mL centrifuge tube at a ratio of 2:1. After centrifugation for 30 min at 3,000 rpm (20 °C), the PBMCs at the plasma-Ficoll interface were collected. Then, PBMCs were washed three times with cold RPMI-1640 medium (containing 5.0% inactivated fetal bovine serum, 0.0599 mg/mL penicillin, 100 μg/mL streptomycin and 24 mM HEPES) by centrifugation at 1,800 rpm for 10 min (4 °C). Cell counts and viability were evaluated using trypan blue staining. The proliferative responses of T cells and B cells after stimulation with ConA (45 μg/mL) and LPS (25 μg/mL), respectively, were determined by MTT assay^61^. ConA, from *Canavalia ensiformis* (C2010), and LPS, from *Escherichia coli* (L2880), were both obtained from Sigma-Aldrich Corporation (Burlington, Vermont, USA). The results are expressed as the SI.

### Peripheral blood lymphocyte classification

PBMCs were isolated using Ficoll density centrifugation. Briefly, heparinized blood was diluted with Hank’s balanced salt solution at a ratio of 1:1 (no calcium, no magnesium, Life Technologies) and layered carefully on the top of Histopaque 1077 (Sigma-Aldrich Corporation) in a 10-mL centrifuge tube at a ratio of 2:1. After centrifugation for 30 min at 3,000 rpm (20 °C), the PBMCs at the plasma-Ficoll interface were collected. Then, PBMCs were washed three times with cold RPMI-1640 medium (containing 5.0% inactivated fetal bovine serum, 0.0599 mg/mL penicillin, 100 μg/mL streptomycin and 24 mM HEPES) by centrifugation at 1,800 rpm for 10 min (4 °C). Cell counts and viability were evaluated using trypan blue staining. Lymphocytes were then mixed with CD3 (SPRD), CD4 (FITC) and CD8 (RPE) antibodies or with Bu-1 (RPE) antibodies. Then, cells were held in a water bath for 30 min (37 °C), washed twice with Hanks solution and fixed with 3% paraformaldehyde. The results are expressed as percentages.

### Antioxidant index measurements

Livers were homogenized with saline to make a 10% homogenate. Alkaline phosphatase and the MT levels in serum samples and the MDA levels; T-SOD, CAT and GSH-Px activities; and T-AOC of the 10% homogenate were determined using a kit (Nanjing Jiancheng Inc., China) according to the manufacture’s protocol.

### Next-generation sequencing (NGS)

Total RNA samples for sequencing were purified from 20 mg of tissue sample collected from 8 chickens (4 chickens in the CON group and 4 in the GEN treatment group) using the RNeasy Fibrous Tissue Mini messenger RNA (mRNA) extraction kit (Qiagen, Hilden, Germany) following the manufacturer’s recommendations. The concentration and purity of total RNA were checked using a UV/Vis spectrophotometer (ACTGene, New Jersey, USA) at 260 nm, and sample integrity was evaluated using a microfluidic assay on a Bioanalyzer system (Agilent Technologies, Inc., Santa Clara, CA, USA). Only high-quality RNA extracts (RNA integrity number (RIN) ≥8) were used for pooling within each treatment group using equal amounts of RNA per chicken. NGS data were obtained from pooled RNA samples within each group to ensure the most robust transcriptome. Construction of complementary DNA (cDNA) libraries for RNA sequencing was performed using a TruSeq RNA Sample Prep Kit v2 (Illumina, San Diego, CA, USA). RNA-Seq analysis was performed for identification of transcriptional changes using a MiSeq instrument (Illumina) according to the manufacturer’s recommendations (CapitalBio, http://cn.capitalbio.com/) using paired-end libraries^62^. Two replicates of each pool were analyzed independent for library synthesis and sequencing. The quality of the raw reads was assessed using FastQC (Version 0.10.1)^63^. Adapters, low-quality reads at the 3′end, reads with fuzzy N bases, rRNA, sequences shorter than 20 nt and low-quality reads (those with a Q < 20) were trimmed with the FASTX clipper (Version 0.0.13). All 126-bp double-end reads of 12 samples from both treatment groups were separately aligned to the chicken reference genome (*Gallus gallus* 5.0, version 81, Ensembl) using the spliced mapping algorithm in TopHat2 (version: 2.0.9)^64^. Unless otherwise stated above, all programs were run with the default parameters. The number of reads equivalent to mapped reads (reads per kilobase per million (RPKM)) was used to normalize the expression of each gene. The quality of obtained data was checked based on the presence and abundance of contaminating sequences, average read length, and GC content. The NGS experiment conformed to the MIAME guidelines^65^, except for the microarray design.

### Bioinformatics analysis

All data processing steps were performed using the Cuffdiff software package (DNASTAR, Madison, WI, USA) for differential gene expression evaluation. Only genes that demonstrated more than a 1.5-fold change in differential expression between dietary groups, with an adjusted P-value ≤ 0.05 from T tests, were selected for further analysis. A Benjamini and Hochberg test (an error rate of 0.05) and an FDR correction test (FDR < 0.05) were used to adjust P-values. Biological mechanisms underlying DEGs were investigated using DAVID v. 6.7 software (the Database for Annotation, Visualization, and Integrated Discovery)^66^ (http://david.abcc.ncifcrf.gov/). The sets of genes were uploaded using official gene IDs. The P-values for the number of genes enriched in biological mechanisms were evaluated using a Benjamini correction, and values less than 0.05 were considered significant. Apart from RNA-Seq, qRT-PCR was performed for individual samples, and a high variation between individual animals was observed. The physiological effects of GEN intake, which were the consequence of the interactions between each animal’s genotype and the treatment, were measured in individual animals. Therefore, it was expected that animal-specific mechanisms would be observed, which were not intended to be the focus of our study. In a pooled-sample experiment, the contribution of these individual-specific reactions is diluted, while the general mechanisms active in all animals remain emphasized. Finally, the molecular interaction networks of DEGs were investigated using the Cytoscape v. 3.1.0 software (http://www.cytoscape.org/)^67^ as a complementary and more comprehensive approach for the identification of central hub genes. The results were visualized using the ClueGO v. 2.1.1. app of the Cytoscape software to create clusters of functionally related genes using the gene ontology databases: KEGG, Wiki Pathways, REACTOME, GO Immune System Process, GO Molecular Function, GO Cellular Component, and GO Biological Process. The probability value was calculated using Enrichment/Depletion (a two-sided hypergeometric test) with Bonferroni correction.

### Statistical analysis

Data are presented as the mean values with their standard deviation and were analyzed by T test with SPSS 18.0 software. Significance was set at P < 0.05, and 0.05 < P < 0.10 was viewed as a trend towards significance.

### Data availability

The datasets generated and/or analyzed during the current study are available from the corresponding author upon reasonable request.
